# A supervised blood vessel segmentation technique for digital Fundus images using Zernike Moment based features

**DOI:** 10.1371/journal.pone.0229831

**Published:** 2020-03-06

**Authors:** Dharmateja Adapa, Alex Noel Joseph Raj, Sai Nikhil Alisetti, Zhemin Zhuang, Ganesan K., Ganesh Naik

**Affiliations:** 1 Key Laboratory of Digital Signal and Image Processing of Guangdong Province, Department of Electronic Engineering, College of Engineering, Shantou University, Shantou, Guangdong, China; 2 TIFAC-CORE, School of Electronics, Vellore Institute of Technology, Vellore, India; 3 MARCS Institute, Western Sydney University, Australia; Sathyabama Institute of Science and Technology, INDIA

## Abstract

This paper proposes a new supervised method for blood vessel segmentation using Zernike moment-based shape descriptors. The method implements a pixel wise classification by computing a 11-D feature vector comprising of both statistical (gray-level) features and shape-based (Zernike moment) features. Also the feature set contains optimal coefficients of the Zernike Moments which were derived based on the maximum differentiability between the blood vessel and background pixels. A manually selected training points obtained from the training set of the DRIVE dataset, covering all possible manifestations were used for training the ANN-based binary classifier. The method was evaluated on unknown test samples of DRIVE and STARE databases and returned accuracies of 0.945 and 0.9486 respectively, outperforming other existing supervised learning methods. Further, the segmented outputs were able to cover thinner blood vessels better than previous methods, aiding in early detection of pathologies.

## Introduction

Digital Eye fundus imaging in ophthalmology plays a significant role in the medical diagnosis of cardiovascular diseases and other pathological conditions such as diabetes and hypertension [1]. These images provide insights into the cardiovascular and ophthalmologic health of patients. However, the manual assessment of these images takes time [2] and requires empirical knowledge [3]. Therefore, automatic detection and analysis of retinal vasculature can help in the screening of diabetic retinopathy, foveal avascular region detection, arteriolar narrowing, premature retinopathy assessment, vessel diameter measurement in relation to hypertension diagnosis and computer-assisted retinopathy [4].

Automated retinal vessel segmentation enhances analysis of the vasculature for screening of various pathological conditions or biometric identification [5]. The challenges faced by segmentation techniques for color Fundus image include intrinsic camera noise from the fundus camera, non-uniform illumination, etc. For the specific case of retinal segmentation techniques, the accuracy is affected by low blood vessel vs. background contrast and irregular shape variations and varying vessel width. Hence, many automated blood vessel segmentation methods have been proposed. Kirbas and F. Quek [6] present a comprehensive study on the extraction of 2D and 3D vessel-like structures in medical images and [7] [8] concentrate on algorithms for automatic detection of diabetic retinopathy in retinal images. Ricci and Perfetti [1] proposed retinal blood vessel segmentation using line operators and support vector machine techniques. Reviews on blood segmentation techniques for retinal images have been presented in [5] [9] with the most recent one being [10].

Existing techniques are either rule-based (with individual pixels being labeled as blood vessel according to pre-defined criteria) or Machine Learning based (use of feature extraction methods). Further, based on the type of segmentation algorithm used, these techniques are further classified as (a) Kernel or Matched Filtering (b) Vessel Tracking methods (c) Morphology and Multiscale approaches (d) Model-based (e) Supervised and (f) Unsupervised methods [10].

Kernel or Matched Filter Response (MFR) techniques [2] [11] employ 2D filter kernels that were tuned to accurately map the profile of the blood vessels within the retinal image. These kernels model the retinal blood vessels through a series of Gaussian shaped filters but tend to accentuate non-blood vessel regions such as red lesions and bright blobs resulting in a degraded performance [10]. Vessel tracking methods [12] [13] [14] capture the blood vessel profiles by tracking or tracing the blood vessels’ central lines obtained by examining the zero-crossing of the gradient function. Morphological techniques [15] [16] employ mathematical transformations such as Top-hat filtering (for enhancement) and Watershed transformation (for segmentation) to identify the vessel profiles in retinal images. Since the width of blood vessels tend to decrease outward from the Optic Disk, Multi-Scale techniques [17] [18] can analyze the geometric and intensity profiles of the blood vessels at various scales to extract the width, size, and orientations for further segmentation. Multiscale techniques are computationally more efficient but present poor segmentation performance for fixed and non-uniform structures like Optic Disk and Retinal Lesions [10]. Model-based techniques [19] [20] extract blood vessel profiles by representing them as flexible curves or surfaces obtained through energy minimization and curve evolution technique. Further techniques mentioned in [21] [22] have provided good accuracies with the standard datasets. A contour detection technique with a clustering algorithm was proposed by [23]. The method required no level set initialization and reported an accuracy of 0.9390 with DRIVE dataset.

Supervised machine learning based Methods rely on prior labeling of classes in which the classifier is trained on a set of manually processed and segmented gold standard (ground truth) references, thereby generating rules for vessel pixel classification. Unsupervised methods classify pixels without any prior labeling information. Preliminary supervised learning models used a back-propagation artificial neural network designed by Nekovei et al. [24] for angiogram images and another by Gardner et al. [25] for vascular tree segmentation. After pre-processing using histogram equalization and edge enhancement, the image was downsized and fed into a Neural Network (NN) with each pixel as a neuron for classifying these pixel windows. Similarly, multilayer perceptron NNs were used later on by Sinthanayothin et al. [26] where pixels were classified using the first principal component and edge weight values of the pre-sub image. These collectively achieved an accuracy of 92%.

For detecting thin blood vessels, unsupervised Deep Neural Networks (DNN) were used by Maji et al. [27], which employed a fusion of deep and ensemble learning for vessel classification via denoising auto-encoder proposed by Roy et al. [28] used on just the trained retinal vascular patches. This approach achieved an accuracy of 93.27%. To improve upon this Lahiri et al. [29] used two parallel auto-encoder ensemble networks where each kernel is responsible for a given orientation of the blood vessel. The first layer was used to train the n-parallel stacked auto encoder NN; the second layer was used to parallelly train the two stacked denoised auto encoders followed by a SoftMax layer at the end. This achieved a high average detection accuracy of 95.3%. This approach, however, wasn’t good in performance and was later improved by Maji et al. in [30] using an ensemble of 12 Convolutional Neural Networks (CNN) with three layers each trained separately using a randomly generated 60000 window patches of size 31x31x31. The results from each layer were then combined to achieve a detection accuracy of 94.7%.

A K-nearest neighbor (kNN) classifier used by Niemeijer et al. [31] featured a 31-component pixel feature vector constructed using Gaussian derivatives of order two at five different scales of the green channel. A supervised ridge-based vessel detection method presented by Staal et al. [32] extracted ridges served as primitives to form line elements to which the pixels are assigned to the nearest line element. A Support Vector Machine (SVM) classifier was used by Ricci et al. [1] which makes use of two orthogonal line detectors with the target pixel gray level to construct the feature vector.

Use of moment invariant features of pixels as part of the feature set for training data is employed by Thangaraj et al. [33], where the 20D feature vector comprises of the maximum Gabor transform responses over different angles, Hu moment invariants, Hessian multiscale filter response, Local Binary Pattern (LBP) of an image and Gray-Level Co-Occurrence Matrix (GLCM) features. Before training the NN, a Principal Component Analysis (PCA) based algorithm was used to reduce the features to a 13D vector, which is used in the NN for training. Focusing on moment invariants, Vaidya et al. [34] have used four different Moment invariants namely, Legendre’s Moment invariants, Zernike’s Moments, Hu Moments and Geometric Moment invariants, to experiment and contrast the performance of different moment invariant techniques. Following this Marin et al. [35] used Hu moment invariants along with grey level features as an input to a NN, trained on manually selected and labelled points of training data from the DRIVE dataset. Selection of the Hu moment invariants was done based on higher performance to load ratio. A 7D (5 grey and 2 Hu moments) feature vector was trained using a basic feedforward network for pattern recognition.

In this paper, we explore a computationally simpler but more effective realization of blood vessel segmentation, inspired by the work of [35]. However, the novelty lines in; (a) use of a superior pre-processing technique to sharpen the blood vessels which were normally performed using a combination of Gaussian and Mean filter, (b) utilization of computationally inexpensive Higher Order, Orthogonal Zernike Moments which can accurately distinguish thinner blood vessel pixels from the background and (c) usage of efficient training set which takes into account all the possible manifestations of the blood vessel, covering the entire training set of the DRIVE database. Compared to other moment-invariant descriptors such as Hu moments, Legendre moments or Geometric moments, Zernike moments offer a higher degree of resolution with its higher and lower order moments. Although we have selected specific Zernike moments, the number of parameters and the combination of all parameters with their possible values posed a challenge for estimation of an optimal set of parameters that will yield the best performance. The intense experimentation required for achieving this demonstrates the rigor of this work.

## Methodology

**Fig 1:**
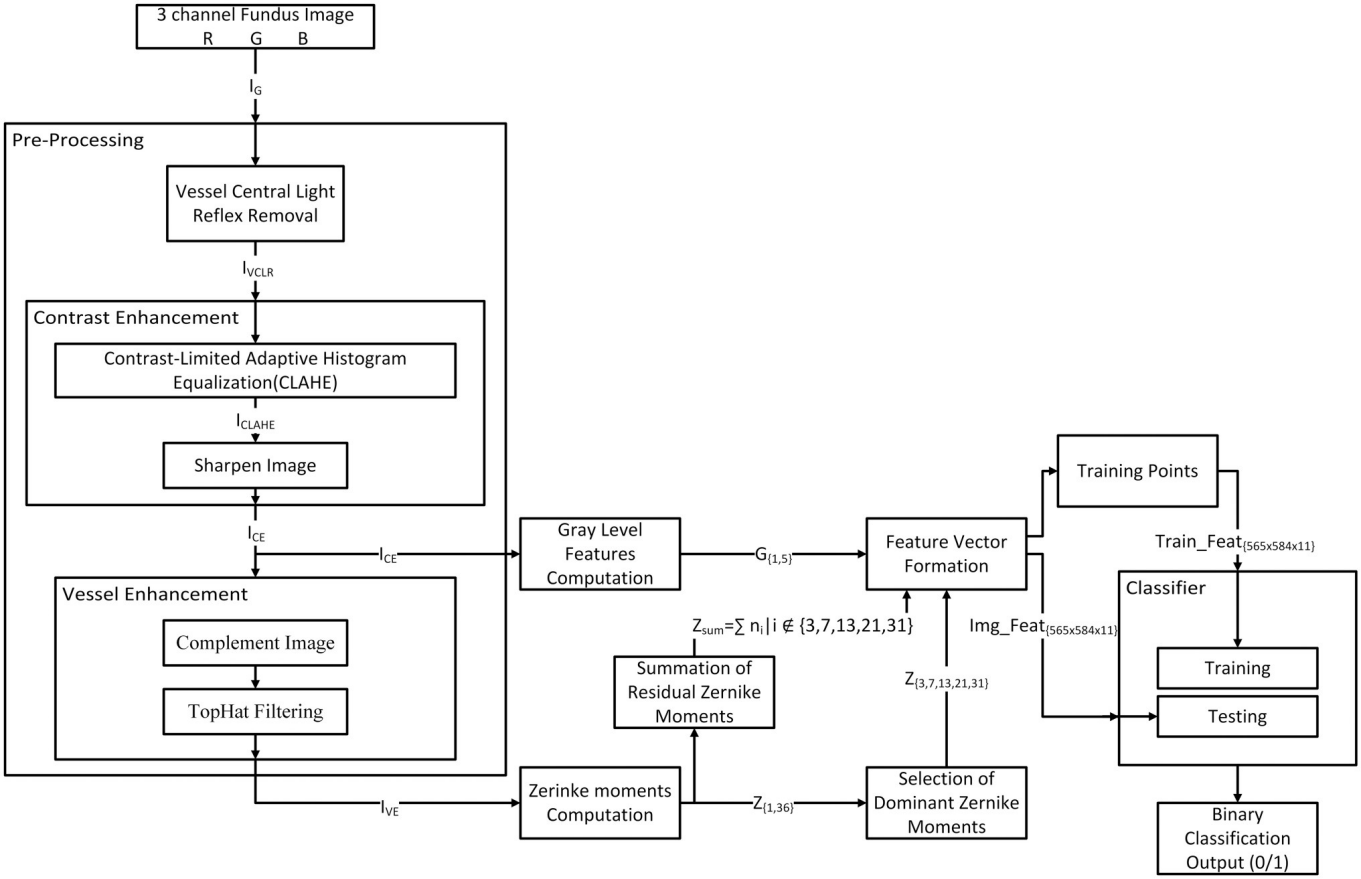
Block diagram representation of the full flow of classification.

The segmentation process can be divided into (a) Pre-processing (b) Feature extraction and selection of dominant features and (c) testing and classification of probable pixels as blood vessels or background. The block diagram of the proposed method is shown in Fig 1.

### Pre-processing

Retinal images are acquired by an ophthalmologist by scanning the retina of the patients using a high resolution fundus camera. Due to the method of scanning, these images show poor contrast between retinal vascular structure and background needing suitable pre-processing techniques to be applied before accurate segmentation of the retinal blood vessels is performed. Numerous techniques have been used in the literature [35] for pre-processing the retinal images. The Green Channel image *I*_*G*_ was used since it contains maximum contrast between background and the blood vessels, to identify central reflex in vessels. The resultant image *I*_*VCLR*_ is obtained by applying the morphological opening operator to the green channel image with a disk-shaped structuring element of diameter 3 pixels. This provides a uniform gray level profile to vessels hence dissolving the central light reflex in the vessels. Since these two procedures have shown to provide satisfactory results [33] [34] [35], we follow the above pipeline.

**Fig 2:**
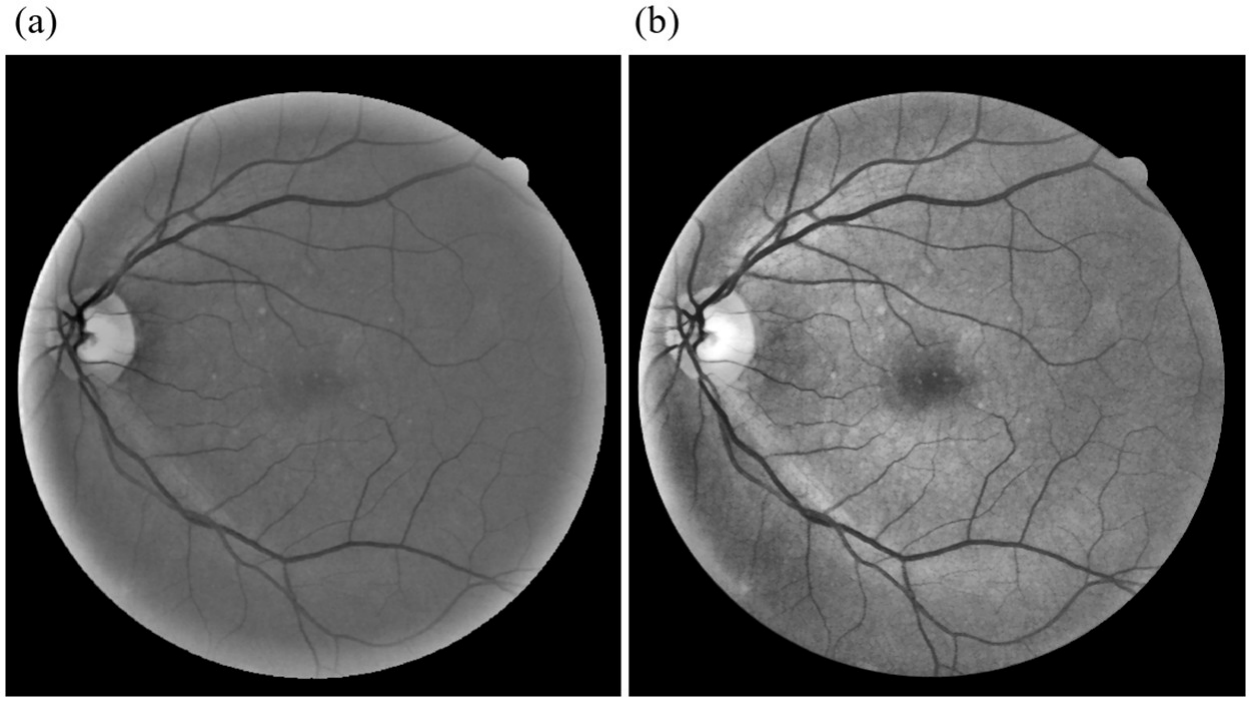
Comparison of Contrast Enhancement between (a)Marin et al. [35] (b) Proposed method through CLAHE.

Non-uniform illumination of the retina causes variations in the intensity leading to poor contrast between blood vessels and their background. This further causes ambiguity in the decision process resulting in a poor segmentation performance [33]. Marin et al. [35] utilized a series of Gaussian and Averaging Filters, but the resulting image loses contrast information for thinner blood vessels. Here we use a Contrast Enhancement technique namely Contrast Limited Adaptive Histogram Equalization (CLAHE) [36] to enhance the contrast based on the local context of the image. CLAHE uses a tile by tile approach to achieve the desired histogram within each tile. We use CLAHE over an 8x8 tile grid to create a flat histogram with the clip limit, and the number of bins was set to 0.01 and 256 respectively over the Vessel Central Reflex Removed Fundus Image. The resulting Enhanced image was qualitatively compared with that of [35] using metrics such as Absolute Mean Brightness Error (AMBE) [37] and Contrast Improvement Index (CII) [38]. The results are summarized in Table 1, and the respective outputs are shown in Fig 2.

**Table 1:** Performance metrics of pre-processing in Marin et al. [35] vs. proposed model.

Performance Metrics	Marin et al. [35]	Proposed Model
Absolute Mean Brightness Error (AMBE) (Lesser is Better)	7.0187	5.5065
Contrast Improvement Index (CII) (Higher is Better)	1.4848	2.3849

It is observed from the values that CLAHE outperforms the background homogenization technique specified in [35] favorably yielding lesser AMBE and higher CII. The Enhanced image *I*_*CLAHE*_ is further sharpened to obtain Contrast Enhanced Image *I*_*CE*_ which is used for generating the vessel enhanced image and the extraction of Statistical Features as explained in *Gray-level Features* section.

**Fig 3:**
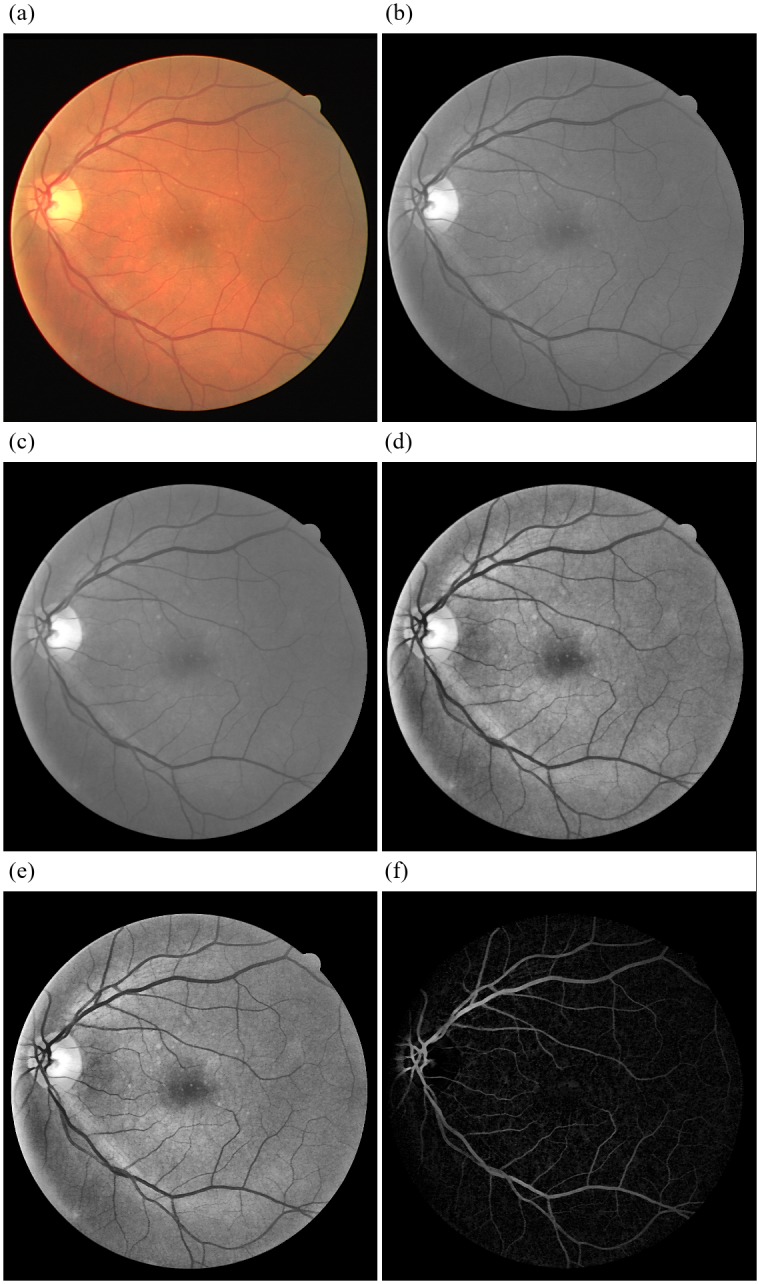
Pre processing for training image 1 from DRIVE dataset. (a)Training Image 1 from DRIVE Dataset (b)Extracted green channel of the RGB Fundus Image *I*_*G*_ (c)green channel image with Vessel Central Reflex Removed *I*_*VCLR*_ (d)CLAHE Applied Image *I*_*CLAHE*_ (e)Contrast Enhanced Image *I*_*CE*_ (f)Vessel Enhanced Image *I*_*VE*_.

To enhance the blood vessel regions from the background, we use the procedure mentioned in [35]. First we compliment *I*_*CE*_, then we apply a morphological Top-Hat transform [39] with a disk-shaped structuring element of diameter 8 pixels. We do this to enhance the blood vessels, remove the Macula (central dark spot) and the Optic Disk (visible as a bright circle where all blood vessels appear to converge). The vessel enhanced image is shown in Fig 3(f). From the figure, it can be seen that *I*_*VE*_ has an enhanced representation of the vasculature suitable for the extraction of shape features as explained in section describing ZM based Features.

The next section explains the extraction of statistical and shape-based features for representing the blood vessels and background pixels in the datasets in the form of quantifiable measurements to be used for classification.

### Feature extraction

Feature extraction is a representation of each pixel of the retinal image in the form of a feature vector that contains meaningful information which can differentiate between the two classes—blood vessels and background. In our method, the feature vector is represented by a set of features consisting of statistical (Gray-Level) features as well as shape-based (Zernike Moments) features.

#### Gray-level features

Gray-Level features are intensity-based and represent the statistical distribution of intensities in the neighborhood of a particular pixel. In Fig 3(e), blood vessels in the Contrast Enhanced image *I*_*CE*_ appear to be darker (have lower gray level) than their surroundings. Hence, features that statistically describe the intensity variations can be used to quantify the presence of blood vessels. Classic Literature suggests the use of mean, standard deviation; and minimum and maximum pixels values within a neighborhood as suitable markers. Hence, we apply a sliding window of size 9x9 pixels with a stride of 1 over the *I*_*CE*_ to calculate the Gray-Level Features as explained in [35]. The size of the sliding window was set based on the maximum width of the blood vessels across all images and all the possible manifestations. The following Gray level features were extracted:

$$\begin{array}{c} G_{1}\left(x,y\right)=I_{CE}\left(x,y\right)-\min_{\left(s,t\right)\epsilon S_{x,y}^{9}}\left\{I_{CE}\left(s,t\right)\right\} \end{array}$$ (1)

where Eq 1 is the first Gray-level feature representing the difference between intensity level at pixel coordinates (x, y) and the minimum intensity within the 9x9 window to enhance the blood vessel with respect to the background.

$$\begin{array}{c} G_{2}\left(x,y\right)=\max_{\left(s,t\right)\epsilon S_{x,y}^{9}}\left\{I_{CE}\left(s,t\right)\right\}-I_{CE}\left(x,y\right) \end{array}$$ (2)

Eq 2 represents the difference between the maximum intensity within the window and the pixel intensity at coordinates (x, y) to enhance the background with respect to the blood vessel.

$$\begin{array}{c} G_{3}\left(x,y\right)=I_{CE}\left(x,y\right)-mean_{\left(s,t\right)\epsilon S_{x,y}^{9}}\left\{I_{CE}\left(s,t\right)\right\} \end{array}$$ (3)

Eq 3 represents the difference between a pixel’s intensity at coordinates (x, y) and mean of all the pixel intensities in the window.

$$\begin{array}{c} G_{4}\left(x,y\right)=std_{\left(s,t\right)\epsilon S_{x,y}^{9}}\left\{I_{CE}\left(s,t\right)\right\} \end{array}$$ (4)

Eq 4 is the fourth Gray-level feature which is the Standard Deviation of all the pixel intensities within the window.

$$\begin{array}{c} G_{5}\left(x,y\right)=I_{CE}\left(s,t\right)\; \end{array}$$ (5)

Eq 5 is the fifth Gray-Level Feature which is the intensity of the Center Pixel of the window. Once the five Gray-Level Features are calculated for each pixel, they are serially concatenated to form the Gray-level feature set as per Eq 6

$$\begin{array}{c} \begin{array}{c} G\left(x,y\right)\;=\;\{G_{1}\left(x,y\right),G_{2}\left(x,y\right),G_{3}\left(x,y\right),G_{4}\left(x,y\right),\\ G_{5}\left(x,y\right)\} \end{array} \end{array}$$ (6)

**Fig 4:**
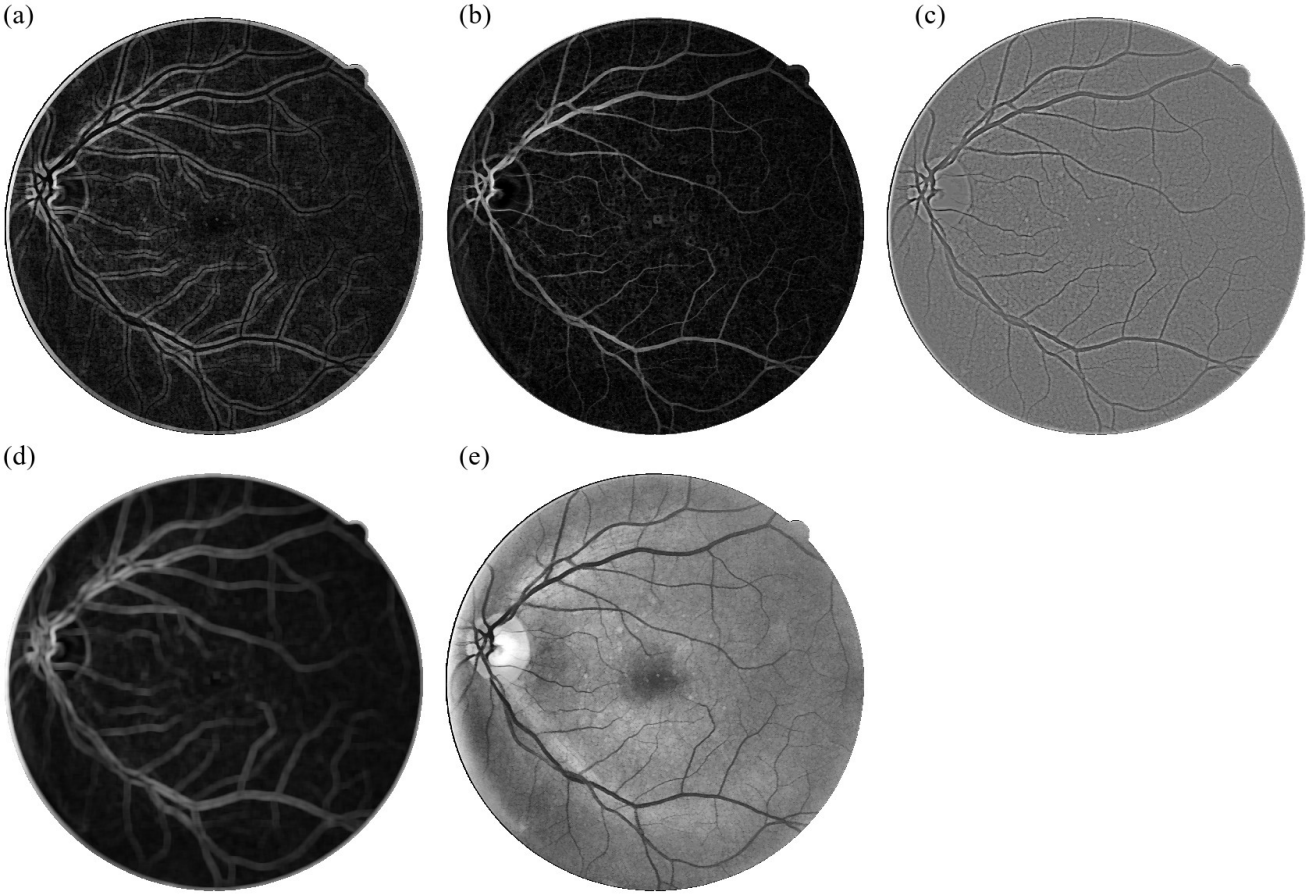
(a) to (f) feature maps of all 5 gray-level features.

Though Gray-Level features statistically represent the presence of blood vessels, they lack in shape information and are sensitive to the Macula and the Optic Disk in the Fundus image as seen in Fig 4. Hence, features describing the shape of the blood vessel are required to provide additional information about the vasculature. The next section describes the extraction of shape features.

#### Zernike Moment (ZM) based features

The vasculature in retinal images exhibits variations in shape, size and geometrical structure as illustrated in Vessel Enhanced Image *I*_*VE*_ in Fig 3(f). A good shape descriptor would help in differentiating the blood vessels from its background. Moment-based descriptors such as Hu moments and ZM have been effectively used for representing the shape parameters [35] [40]. The desirable characteristics of a shape descriptor are (a) invariance to change in rotation, scale and translation, (b) provide features with low redundancy and large discrimination ability and (c) present hierarchical representation, i.e., furnish coarse (global) to finer (local) details. Literature indicates that ZM [41] due to their orthogonality, have the lowest feature redundancy, and it is also observed that their hierarchical nature allows the lower order moments to provide global information, and higher-order moments to provide local information respectively [40]. In contrast, Hu Moments are Algebraic, hence have higher feature redundancy. Also, it is computationally more expensive to compute the higher order Hu moments. Therefore, we use the ZM based features for representing the shape features of the blood vessels. For the calculation of ZMs, a window of 17x17 was extracted. This arbitrary size was chosen so that the region is centered on the middle of a wide vessel around 8-9 pixels wide on retinal fundus images of approximately 540 pixels in diameter. The sub-image thus obtained is shown to contain an approximately equal number of the vessel and non-vessel pixels similar to the pre-processing work done by [35]. The images of DRIVE dataset on which the model is trained, have a standard 540 pixels diameter. Thus, when working with data obtained from new sources with higher resolution or larger imaging, it is required to rescale the input images accordingly. The extracted window of 17x17 pixels is then multiplied by a Gaussian kernel of the same size to yield an active region of 9x9 pixels in the center of the window as given in Eq 7.

$$\begin{array}{c} S\left(x,y\right)=I_{VE}\left(x,y\right)*G\left(x,y\right) \end{array}$$ (7)

here G(x,y) is the Gaussian kernel of size 17x17 pixels with (mean)*μ* = 0 and (standard deviation)*σ* = 1.65. The multiplication process provides (a) substantial reduction of noise within the chosen image window and (b) solves a greater problem of accurate classification and localization of the center pixel of blood vessel within the 17x17 window as stated in [35]. The mean and standard deviation of the Gaussian kernel are selected after explicit experimentation to obtain the maximum coverage of Gaussian distribution. By using these values paired with a window size of 17x17, we are able to contain 98% of the area represented by the Gaussian distribution and the remaining values being close to 0. The above process helps us to determine whether the pixel is at the center of the blood Vessel or the boundary. Next, we compute the ZM, by projecting S(x, y) on to a set of complex Zernike polynomials as given in Eq 8.

$$\begin{array}{c} A_{nm}=\frac{m+1}{\pi }\int_{X}\int_{Y}S\left(x,y\right)V_{nm}^{*}\left(\rho ,\theta \right)dxdy,x^{2}+y^{2}\leq 1 \end{array}$$ (8)

With

$$\begin{array}{c} V_{nm}\left(x,y\right)=V_{nm}\left(\rho ,\theta \right),R_{nm}\left(\rho \right)e^{jm\theta } \end{array}$$ (9)

Where, *V*_*nm*_(*x*, *y*) is the Zernike Polynomial, *S*(*x*, *y*) is a window of 17x17 pixels from Eq 7, n is the order of the polynomial, m is the repetition factor such that |*m*| ≤ *n* and *n* − |*m*|is even, *ρ* is the length of the vector from the origin to the pixel located at the spatial location (x, y) and is given by $\rho =\sqrt{x^{2}+y^{2}}$, *θ* is the angle of the vector from the origin to the pixel located at the spatial location (x, y) from the x-axis in a counter-clockwise direction and *R*_*nm*_ is the radial polynomial defined as:

$$\begin{array}{c} R_{nm}(\rho )\;=\;\sum_{s=0}^{n-|m|}(-1{)}^{s}\frac{(2n+1-s)!}{s!(n-|m|-s)!(n+|m|+1-s)!}\rho^{n-s} \end{array}$$ (10)

The ZM is obtained from the equation is a complex quantity given by:

$$\begin{array}{c} A_{nm}=R_{ZM}+jI_{ZM} \end{array}$$ (11)

$$\begin{array}{c} |A_{nm}|=\sqrt{R_{ZM}^{2}+I_{ZM}^{2}} \end{array}$$ (12)

**Fig 5:**
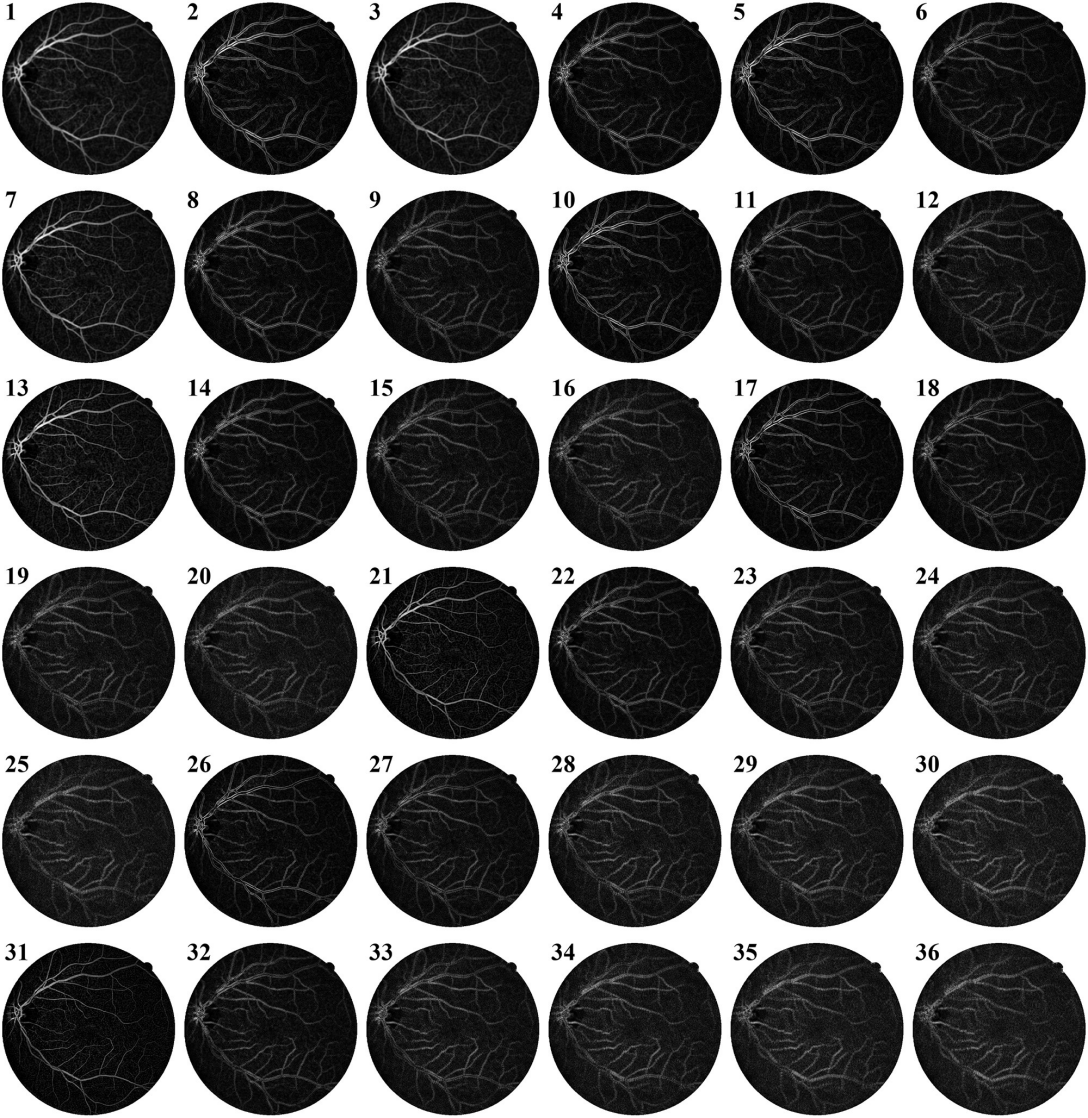
Zernike Moments feature maps for training image 1 from DRIVE dataset.

In Eq 12, |*A*_*nm*_| represents the shape descriptor feature obtained from ZM for specified order n and repetition factor m. A total of 36 such ZM with n ranging over 1 to 10 and corresponding combinations of n, m with m such that |*m*| ≤ *n*, *m* ≥ 0 and *n*−|*m*| is even, are calculated for the center pixel of the window each sliding window. Subsequently, the process is applied on *I*_*VE*_ with a stride of 1 pixel along the row and column to obtain Zernike feature maps as shown in Fig 5.

**Fig 6:**
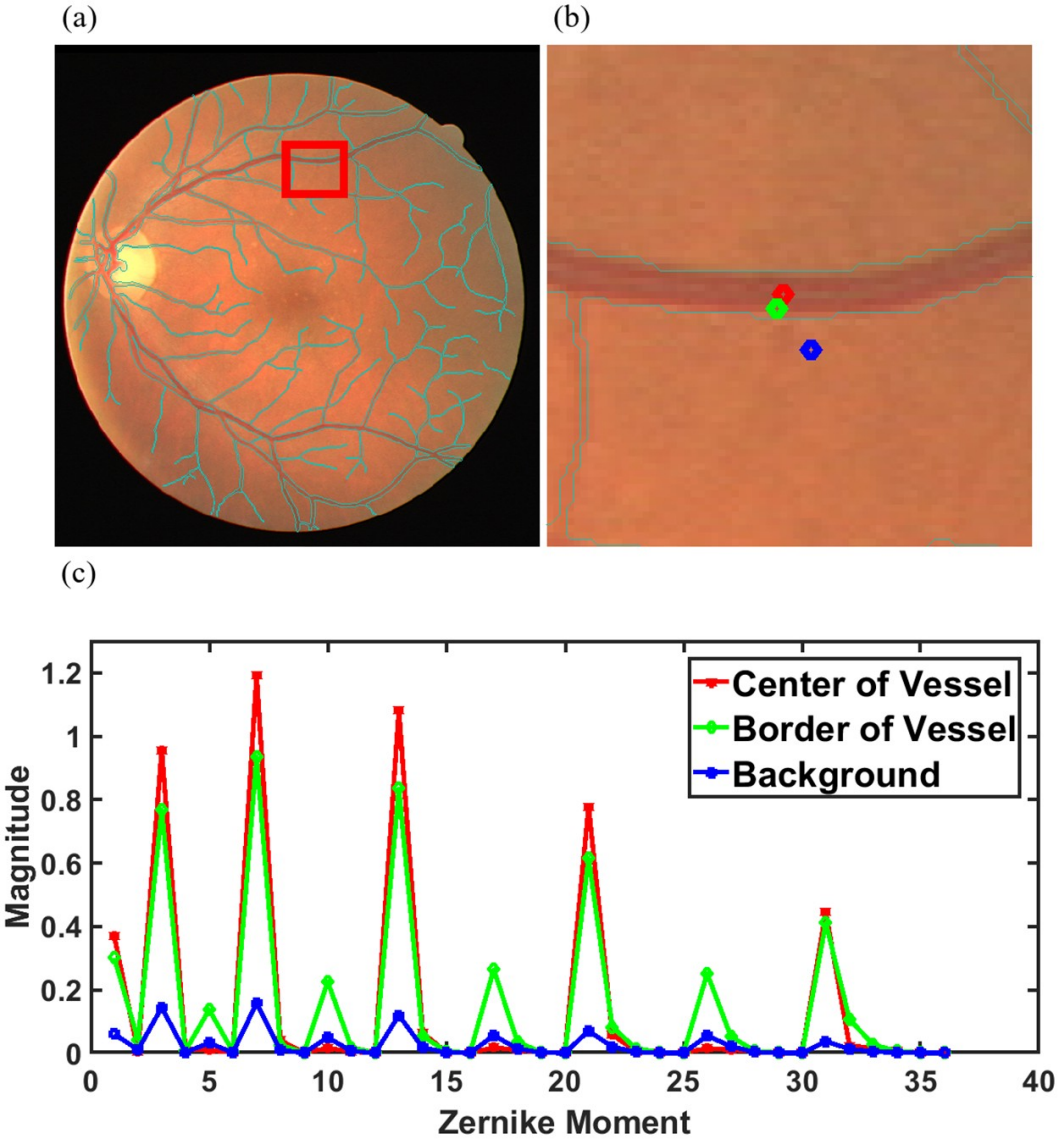
Selection of Dominant Zernike Moments. (a) Selected Section in Training Image 1 from DRIVE dataset, (b) Pixel in Center of blood vessel (Red), Boundary of Blood Vessel (Green), Background Pixel (Blue), (c) Zernike Moment Graphs for the three selected pixels.

As observed from the ZM feature maps in Fig 5, all ZM coefficients do not give a very accurate representation of the blood vessels present in the vessel enhanced image. Also, having 36 Zernike features in addition to 5 gray-level features representing each pixel will lead to an unnecessary increase in computational complexity during classification. Thus, we select the ZMs which can accurately differentiate the blood vessels from the background. Dominant Zernike Moments (DZM) are a subset of all 36 ZMs that provide the maximum variation between a blood vessel and background pixel for aiding segmentation. For selecting the DZM, we first observe the values of 36 Zernike Moments calculated over three types of pixels as shown in Fig 6(b)—pixel in Center of Vessel (red), a pixel on Border of Vessel (green) and Background pixel (blue).

It is observed from Fig 6(c) that magnitude of 3^*rd*^, 7^*th*^, 13^*th*^, 21^*st*^ *and* 31^*st*^ ZM at the center as well as the boundary of the blood vessel showed considerable difference when compared to corresponding ZM for the background pixel. These correspond to the ZM: |*A*_2,0_|, |*A*_4,0_|, |*A*_6,0_|, |*A*_8,0_|, and |*A*_10,0_|.

**Fig 7:**
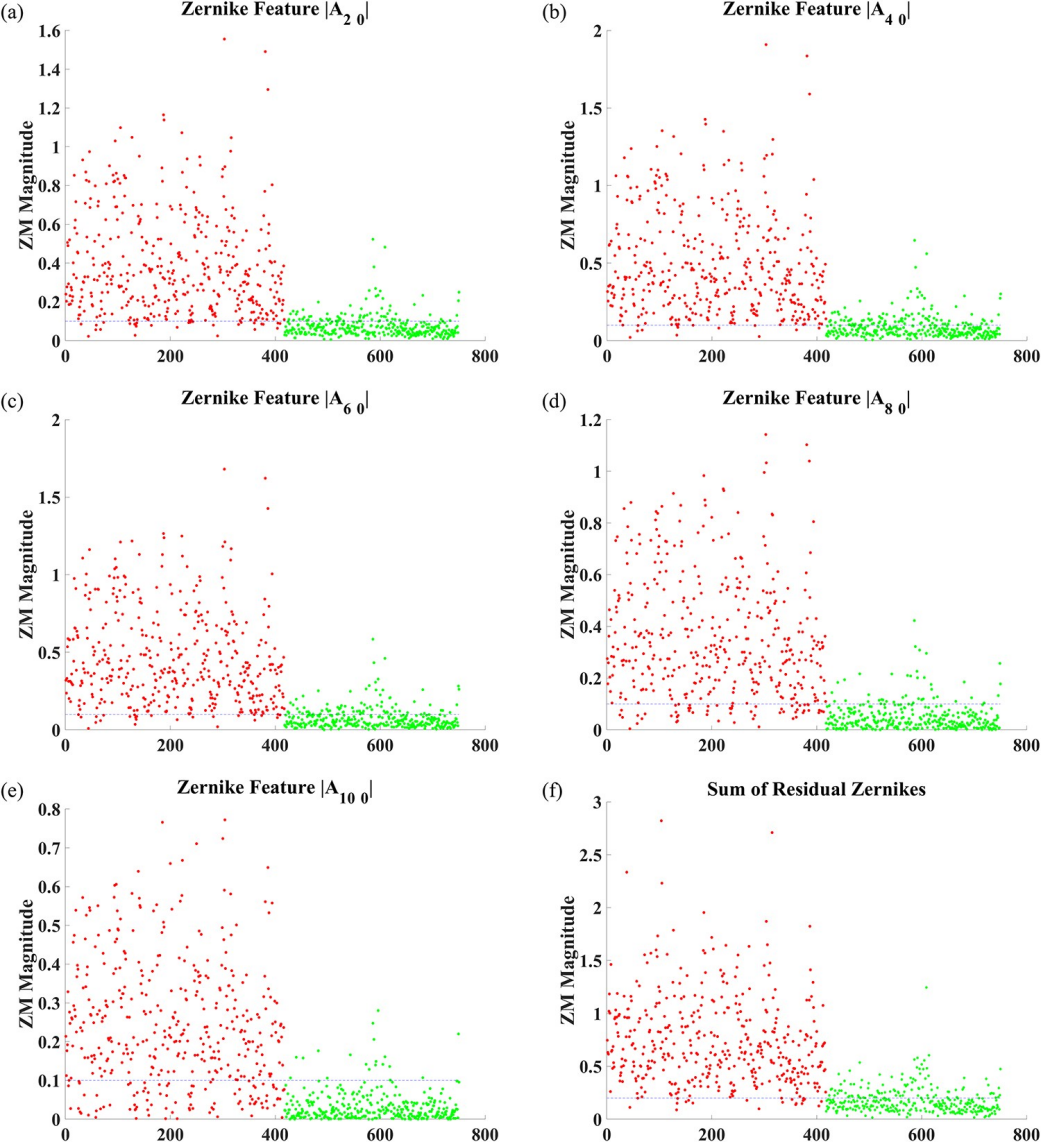
Plots of magnitudes for individual DZM features. Red dots represent blood vessel Pixels, and Green Dots Represent background pixels.

To further substantiate that the selected ZM have maximum variation between the blood vessel pixels and background pixels, we plot (refer Fig 7) the magnitudes of the selected ZM for a set of pixels for retinal image 21 from DRIVE dataset. Each graph in Fig 7 represents the selected pixels along the x-axis and ZM magnitude on the y-axis. Here the red dots represent blood vessel (center or boundary), and green ones represent background pixels respectively. It can be observed that there is a clear distinction with respect to the magnitude of the ZM with the background pixels densely packed at the bottom and the blood vessel pixels mostly staying above a particular threshold. Therefore, these five moments, categorized as Dominant Zernike Moments (DZM) together help classify the pixels as blood vessels or not. The DZM Feature Set for each pixel is given as:

$$\begin{array}{c} DZM=\{|A_{2,0}|,|A_{4,0}|,|A_{6,0}|,|A_{8,0}|,|A_{10,0}|\} \end{array}$$ (13)

Zernike Moments being orthogonal, present additional information for each combination of *A*_*nm*_. Having bigger feature vector by appending all ZM will attribute to providing equal weightage to all features which may result in a) misclassification of blood vessel pixels and b) lowering the contributions of DZM. Therefore, to prevent loss of information, we sum the remaining ZM which are not part DZM and append them as additional shape descriptor as given in Eqs 14 and 15.

$$\begin{array}{c} Z_{s}=\sum_{l=1}^{l=36}Z_{\left\{l|l\notin \{3,5,7,13,21,31\}\right\}} \end{array}$$ (14)

$$\begin{array}{c} Z=\{DZM,Z_{s}\} \end{array}$$ (15)

**Fig 8:**
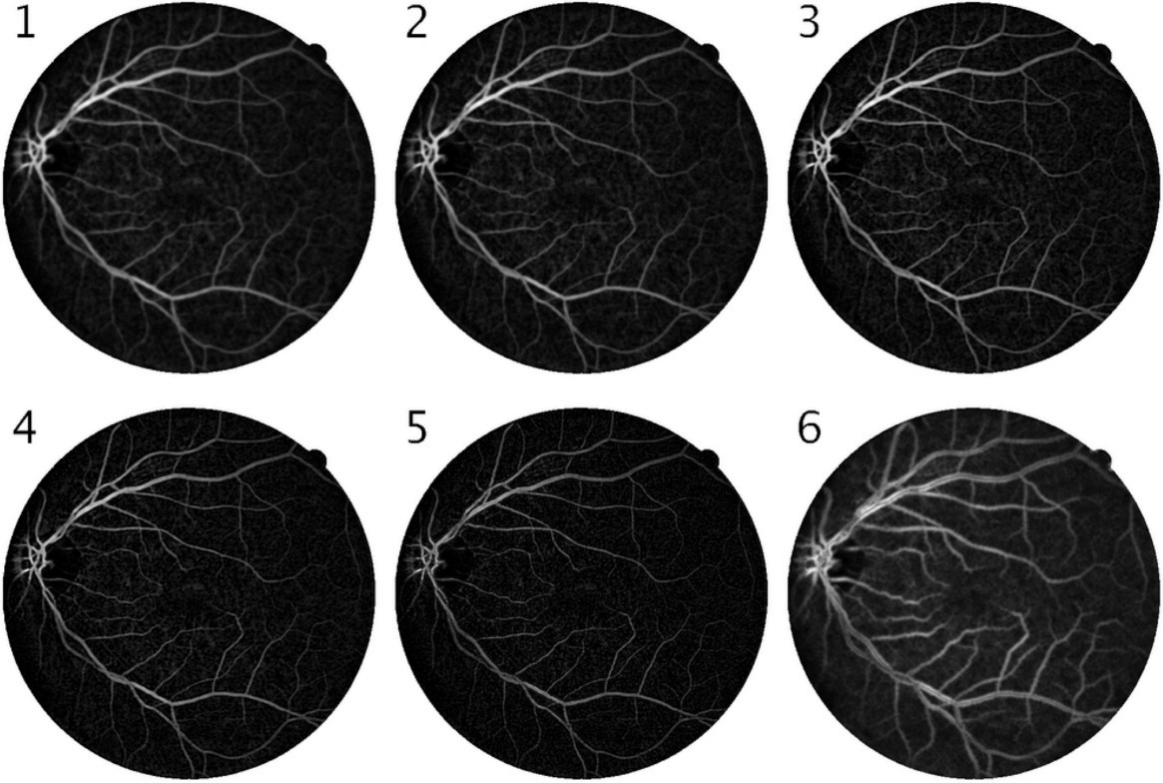
Dominant Zernike Moment feature maps (first five images) and summation of residual Zernike feature maps (last image) for training image 1 from DRIVE dataset.

Where *Z*_*s*_ represents the summation of the residual Zernike moments. It can also be seen from Fig 7(f) that *Z*_*S*_ also provides a clear distinction between the blood vessel pixels and the background. The feature maps for the DZM and *Z*_*s*_ for the Image 1 of the DRIVE Dataset is shown in Fig 8.

### Feature vector representation

Summarizing all the features discussed in the previous section, a feature vector is generated for each pixel by concatenating the statistical 6 and shape features 15. Thus, features are ordered as:

$$\begin{array}{c} f_{i,j,l}=\left\{G_{i,j},Z_{i,j}\right\} \end{array}$$ (16)

here, *f*_*i*,*j*,*l*_ is the feature vector of the pixel at coordinates (i, j) and l ranges from 1 to 11 accounting for the combined set of 11 features, *G*_*i*,*j*_ represents the five Gray-level features for the pixel at coordinates (i, j) using Eq 6, *Z*_*i*,*j*_ represents the five ZM based features for the pixel at coordinates (i, j) using Eq 15.

Further, the feature vector is normalized as per Eq 17

$$\begin{array}{c} f_{i,j}=\frac{f_{i,j}-\mu_{i}}{\sigma_{i}} \end{array}$$ (17)

where *μ*_*i*_ and *σ*_*i*_ are the mean and standard deviation of all the features in for the pixel at coordinates (i, j).

## Classification using ANN

**Fig 9:**
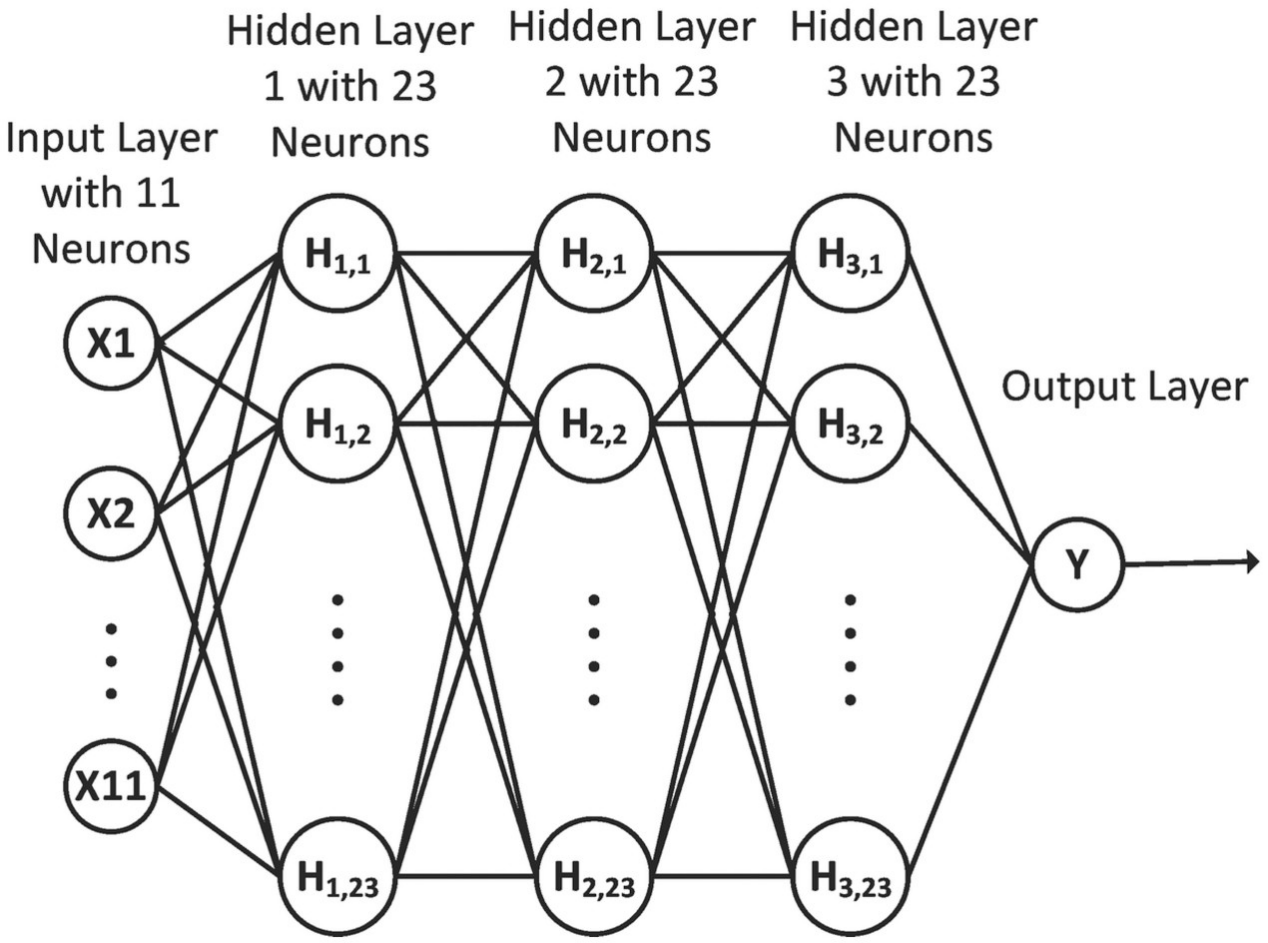
Architecture of the ANN.

The Set of Features obtained in the *Feature Extraction Section* represents the Gray-Level Statistics and the shape descriptors of each pixel within the FOV of every image for both the datasets. A supervised learning approach is implemented by training an Artificial Neural Network (ANN) based binary classifier. The architecture used is similar to the multi-layer feed forward network in [35] with the difference being that the input layer size has changed in accordance with the number of input features we used (11 features) and each of the three hidden layers has 23 neurons (refer Fig 9). The neural network consists of 1357 total learnable parameters, 253 (11x23) in the 1^st^ layer, 529 (23x23) in the 2^nd^ layer, 529 (23x23) in the 3^rd^ layer and 46 (2x23) in the final layer. These parameters or weights of the network are modified during training. All the three hidden layers have a tan-sigmoid activation function. The output layer is a soft-max layer that gives two probability outputs with a first output representing the probability of being a blood vessel pixel and the second output being complementary to the first output and representing the probability of the pixel is a background pixel. The loss function is set to cross-entropy loss with the training function being Scale Conjugate Gradient (SCG) descent.

## Design of experiments

### Datasets

The algorithm was evaluated on two publicly available manually annotated Fundus Image Datasets—DRIVE (Digital Retinal Images for Vessel Extraction) [32] and STARE (STructured Analysis of the Retina) [2]. These two datasets have found a mention for their use in benchmarking most of the retinal vessel segmentation methodologies [5] [17] [35] [42] [43] [44]. The DRIVE database consists of 40 color Fundus Images distributed equally across a training set and testing set. The database provides ground truth images formed by manual annotation for all the 40 images with two sets of annotations for the testing set. Along with the color images and ground truth annotations, the Field-of-View (FOV) mask is also provided. The STARE database contains a total of about 400 images. Out of these, 20 color Fundus Images with two sets of ground truth images for blood vessel Segmentation is provided. For comparison with the rest of the methods, we use the ground truth provided by Adam Hoover [2]. There is no FOV mask provided. Hence, the FOV masks were generated by hand using a photo editing tool.

### Training dataset

Fundus Images contain more of background pixels than vessel pixels. Using all the pixels in the image as training data will result in a skewed dataset with the classifier being able to classify background pixels better than the vessel pixels with visible divergence during testing of the neural network. Hence, a training dataset was required with the acceptable distribution of vessel and background pixels along with coverage of all possible manifestations of blood vessels over different illumination levels, different background contrast levels and varying shapes of the Optic Disk and Macula.

To have a comprehensive comparison we first used the training points provided in [35]. This contained points with vessel pixels and background pixels spread across images 21, 22, 25, 30 and 31 from the training set of the DRIVE dataset, but training results provided an overall average accuracy of 0.84. However, as discussed in [35], thinner blood vessels were not picked up by this classifier leading to reduced overall sensitivity.

**Fig 10:**
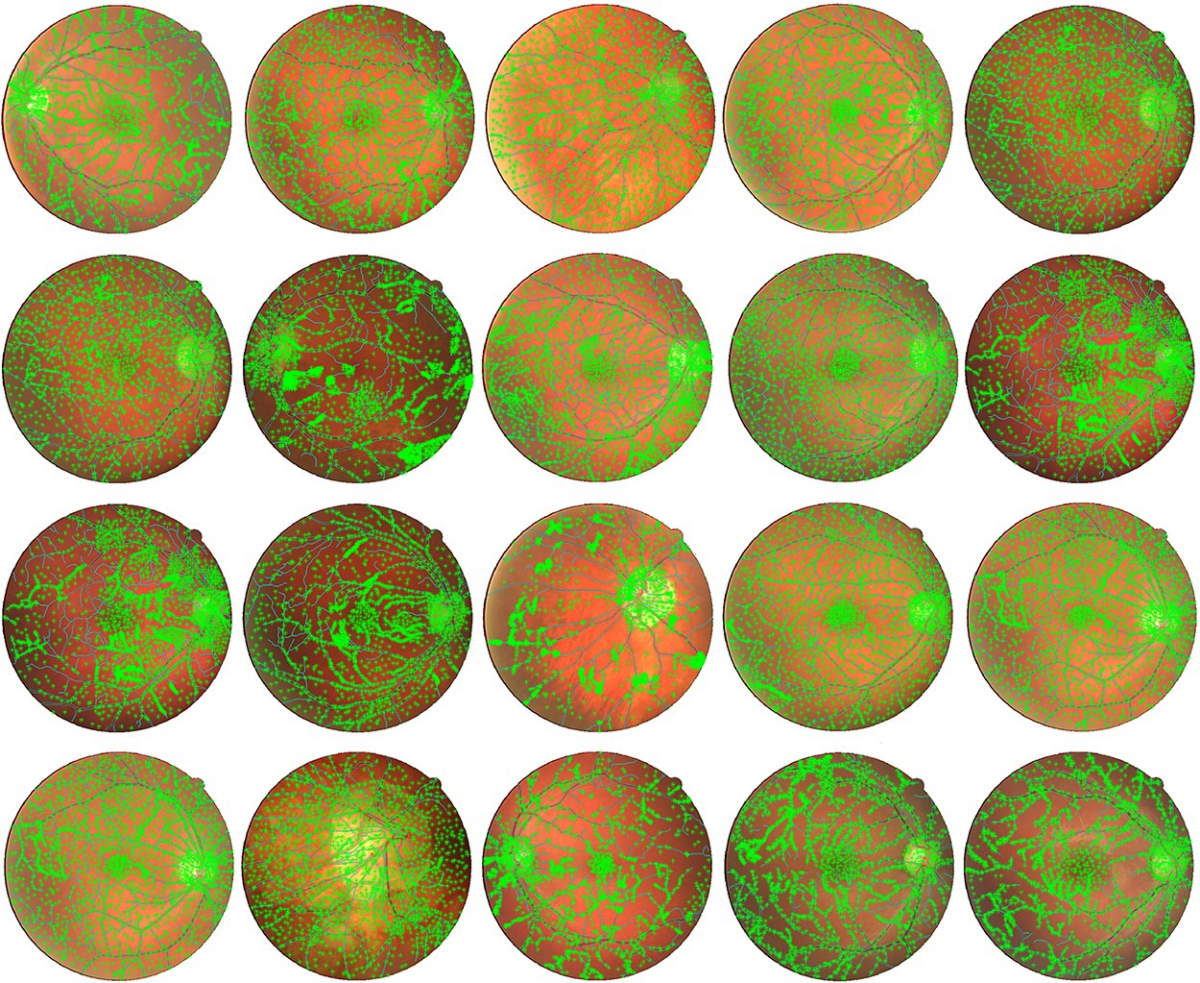
Manually selected training points from a training set of DRIVE database.

It was observed that the training set did not contain points from single blood vessels and locations near the Optic Disk and Macula. The performance error of the trained network was also getting saturated at 0.18 without reaching convergence. Therefore, a MATLAB based Graphical User Interface (GUI) was developed to help manually select all patterns of blood vessels. The final set of training points consisted of 34609 points with 15305 blood vessel Pixels and 19304 background pixels with each training image having almost 1730 points. The greater number of background pixels is due to picking up more points on the Macula and Optic Disk to avoid misclassification of these features as blood vessels. Fig 10 shows the training points selected from the DRIVE dataset.

For training the ANN, for each pixel, the feature vector is formed as per Eq 16 and presented to the input of the ANN along with target binary classification of the pixel. The targets are given to the ANN in the form of Eq 18:

$$\begin{array}{c} T_{i}=\left[C1_{i},C2_{i}\right] \end{array}$$ (18)

Where ‘i’ refers to the training set points. The target for a pixel to be classified as a blood vessel is given as C1 = 1 and C2 = 0 and for a pixel to be classified as the background is given as C1 = 0 and C2 = 1.

We conducted a set of experiments exploring different combinations of features for training the ANN Classifier. Table 2 illustrates the performance of the different combinations of ZM features. It was verified that the feature set comprising of the 5 Gray Level, 5 DZM and the sum of the Residual ZMs presented the best error performance of 0.0218 after 3603 epochs requiring 4 minutes and 35 seconds when trained on ANN. Further, the performance of the selected features was quantified by commonly used performance measures. The confusion matrix for the same has been presented in Table 3.

**Table 2:** Performance of combinations of features.

Feature Set	Accuracy	Error
5G+5log(Z)	0.974	0.308
5G+5Z	0.978	0.262
5G+5Z+sum(5Z)	0.98	0.25
5G+5Z+sum(Res(Z))	0.982	0.0218

**Table 3:** Confusion matrix of training data for the best feature set.

		Target Class		
		Blood Vessel	Background	Accuracy
Output Class	Blood Vessel	15008 (TP) 43.4%	297 (FN) 0.9%	98.2%
	Background	325 (FP) 0.9%	18979 (TN) 54.8%	

## Experimental analysis

For testing the classifier, a total of 4537859 points spread across 20 images for the DRIVE testing set and 6207314 points spread across 20 images of the STARE Blood vessel segmentation dataset. All the experiments were performed using Matlab 2017a running on a workstation with 2 x Intel Xeon E2620 v4 CPU, 64GB RAM, and Nvidia Tesla K40 GPU. Feature Generation takes around 17 minutes for a set of 10 images using optimized parallel programming effort. The time for training the NN using the GPU was a reduced 5 mins. The testing time for a batch of 20 images is around 3 mins.

**Fig 11:**
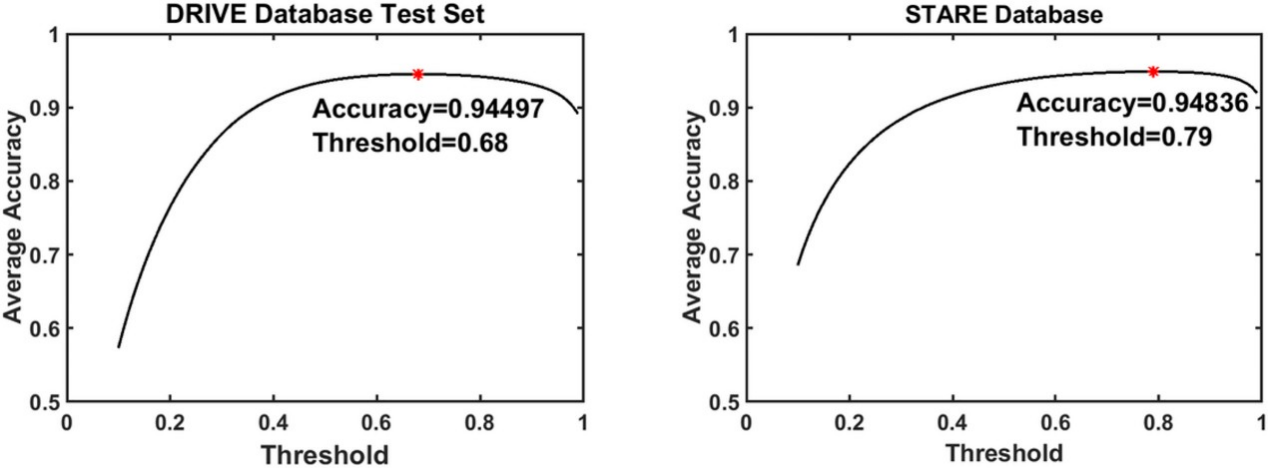
Threshold vs accuracy plots. (a) DRIVE and (b) STARE database Threshold Vs. Accuracy Plot.

As explained earlier, the feature vector comprising of 11 features is passed to the ANN as input. The output of the ANN Classifier is a probability score for each of the classes (blood vessel and background). To assign a particular pixel to one of these classes, a threshold is applied over the probability score of the blood vessel class. Different thresholds give varying accuracy results. Hence, we obtain the optimal threshold (*T*_*optimum*_) value for the entire dataset by considering the overall accuracy obtained from the testing data by increasing threshold between 0 and 0.99 and selecting the threshold that provides maximum accuracy. The threshold vs. accuracy plots for the DRIVE and STARE databases is shown in Fig 11(a) and 11(b) respectively. From the graphs, it has been determined that the *T*_*optimum*_ for the DRIVE and STARE databases are 0.68 and 0.79 respectively.

**Fig 12:**
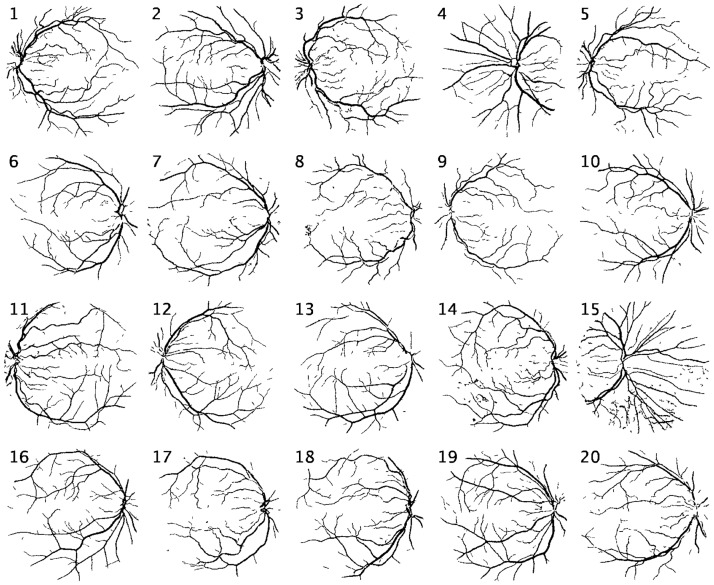
DRIVE database segmentation results.

**Fig 13:**
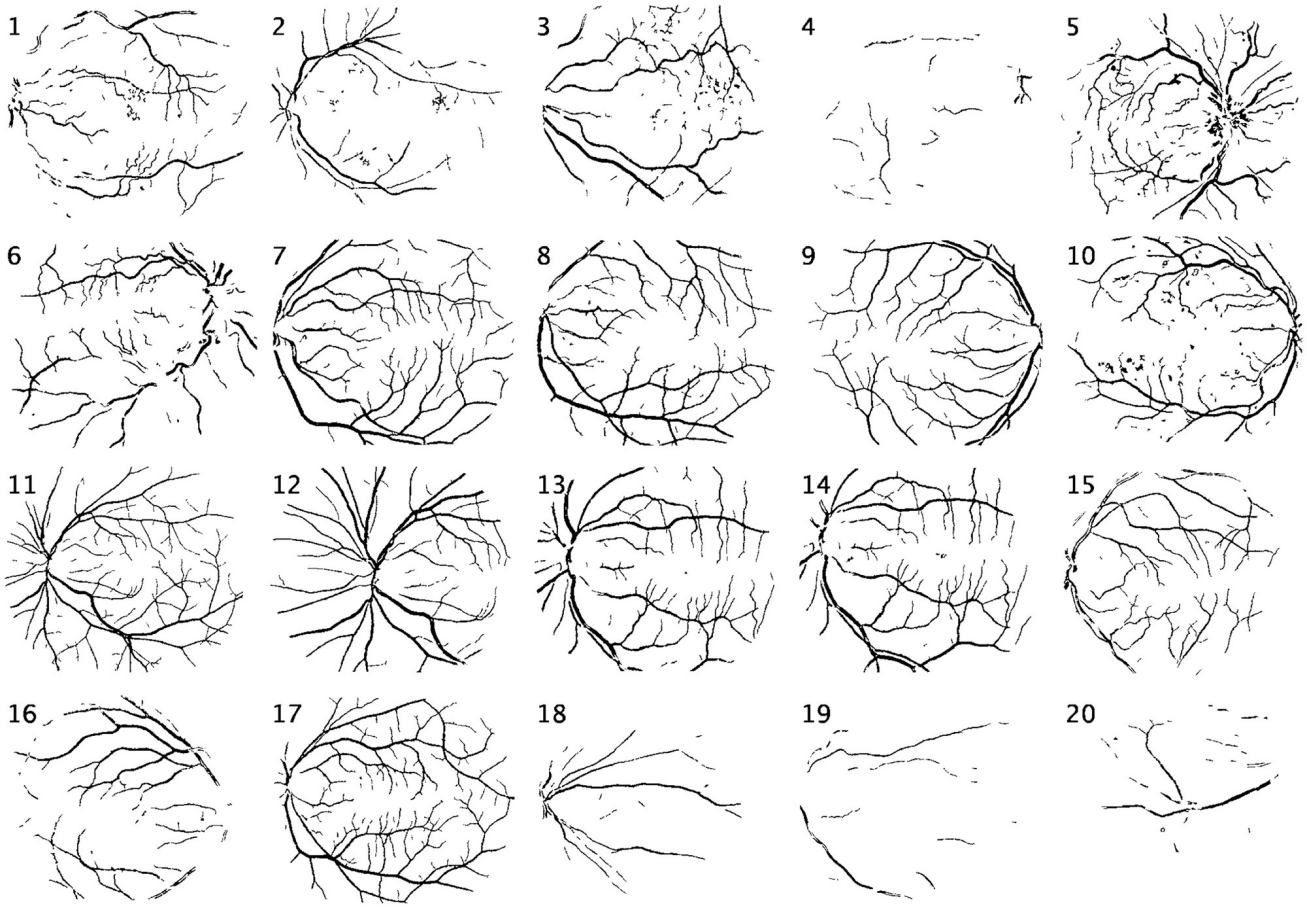
STARE database segmentation results.

**Fig 14:**
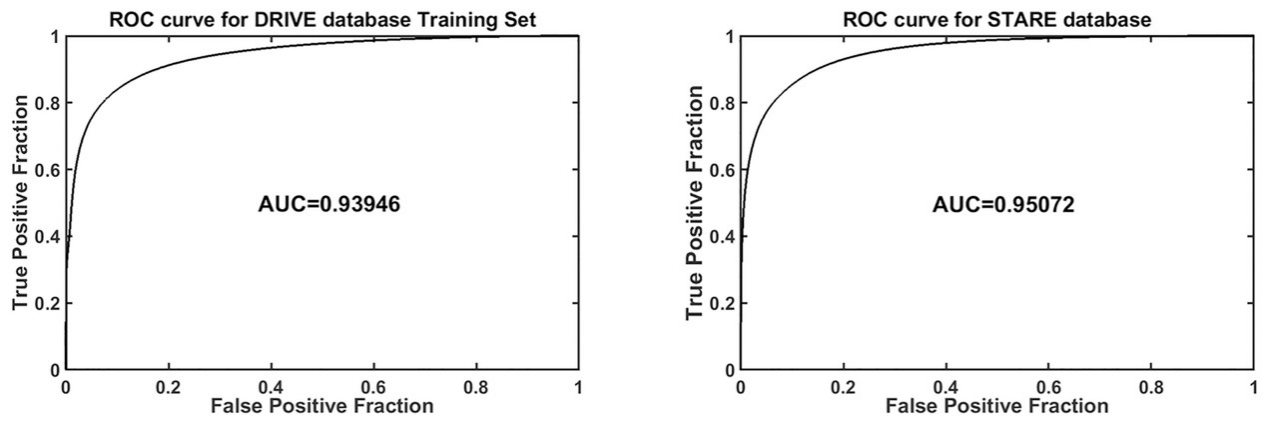
ROC curve for (a) DRIVE and (b) STARE database testing.

The blood vessel segmented images for DRIVE and STARE datasets are shown in Figs 12 and 13 respectively. Similarly, the performance measures for the individual test images in Tables 4 and 5. Further, the ROC curves obtained from the classification of the testing data are presented in Fig 14.

**Table 4:** Performance measures for neural network tested on DRIVE database.

Image No.	Accuracy $\frac{TP+TN}{TP+FP+TN+FN}$	F1_Score $\frac{2(Precision*Recall)}{Precision+Recall}$	False Positive Rate $\frac{FP}{TN+FN}$	Precision $\frac{TP}{TP+FP}$	Sensitivity $\frac{TP}{TP+FP}$	Specificity $1-\frac{FP}{TN+FN}$
1	0.9492	0.7984	0.0235	0.8316	0.7677	0.9765
2	0.9482	0.8135	0.0175	0.8835	0.7538	0.9825
3	0.9364	0.7601	0.0217	0.8447	0.6908	0.9783
4	0.9477	0.785	0.0166	0.8691	0.7157	0.9834
5	0.9443	0.7667	0.0132	0.8888	0.6741	0.9868
6	0.9349	0.7211	0.0095	0.9115	0.5965	0.9905
7	0.9439	0.7629	0.0161	0.8661	0.6816	0.9839
8	0.9398	0.7169	0.0124	0.8759	0.6068	0.9876
9	0.9427	0.6986	0.0071	0.914	0.5654	0.9929
10	0.9491	0.7683	0.018	0.842	0.7064	0.982
11	0.9414	0.7606	0.0252	0.8092	0.7174	0.9748
12	0.9469	0.7667	0.0174	0.8515	0.6972	0.9826
13	0.9368	0.7363	0.0113	0.9007	0.6226	0.9887
14	0.9466	0.7705	0.0284	0.7814	0.7599	0.9716
15	0.9345	0.7255	0.0538	0.6422	0.8337	0.9462
16	0.9491	0.7825	0.0134	0.8872	0.6998	0.9866
17	0.9422	0.7215	0.0107	0.8884	0.6074	0.9893
18	0.9499	0.766	0.0194	0.8267	0.7136	0.9806
19	0.9612	0.8388	0.0222	0.8381	0.8394	0.9778
20	0.9543	0.7749	0.0198	0.8162	0.7376	0.9802
Average	0.945	0.7617	0.0189	0.8484	0.6994	0.9811

TP = True Positives, TN = True Negatives, FP = False Positives, FN = False Negatives

**Table 5:** Performance measures for neural network tested on STARE database.

Image No.	Accuracy	F1_Score	False Positive Rate	Precision	Sensitivity	Specificity
1	0.9384	0.6543	0.0128	0.8363	0.5374	0.9872
2	0.948	0.6468	0.0094	0.8484	0.5226	0.9906
3	0.9557	0.7259	0.0236	0.7298	0.7221	0.9764
4	0.9118	0.241	0.0004	0.9773	0.1374	0.9996
5	0.9356	0.7266	0.0308	0.7602	0.6959	0.9692
6	0.9625	0.7767	0.0167	0.8109	0.7453	0.9833
7	0.9576	0.8166	0.031	0.7737	0.8646	0.969
8	0.9596	0.8116	0.0282	0.7745	0.8524	0.9718
9	0.9611	0.8154	0.0195	0.8317	0.7997	0.9805
10	0.9463	0.7524	0.0288	0.7611	0.7439	0.9712
11	0.9607	0.8063	0.0264	0.7741	0.8414	0.9736
12	0.9635	0.8352	0.0265	0.7958	0.8787	0.9735
13	0.9554	0.8054	0.0174	0.8584	0.7587	0.9826
14	0.956	0.8084	0.0145	0.8798	0.7478	0.9855
15	0.9377	0.668	0.0085	0.8929	0.5336	0.9915
16	0.9184	0.6041	0.0048	0.9381	0.4455	0.9952
17	0.9591	0.8262	0.0178	0.8611	0.7941	0.9822
18	0.9621	0.6339	0.0016	0.9553	0.4743	0.9984
19	0.9572	0.4518	0.0017	0.9173	0.2997	0.9983
20	0.9253	0.3279	0.0022	0.9023	0.2004	0.9978
Average	0.9486	0.6867	0.0161	0.8439	0.6298	0.9839

It is observed in Fig 13 that the segmented vasculature of images 4, 19 and 20 appear to be incomplete. It should be noted that the original test image 4 of the STARE dataset is acquired under low lighting conditions and hence has poor contrast between the blood vessels and the background. Similarly in test images 19 and 20, the thin blood vessels are not easily distinguishable.

## Comparison with previous methods

We compare our technique with methods that employ supervised classification using ANN for blood vessel segmentation. In [35], algebraic Hu Moments were used as shape descriptors along with gray scale features whereas [33] (which is one of the latest publications) employs a 13 dimensional feature vector comprising of Gabor filter responses, Frangi’s vesselness measure, Local Binary Pattern (LBP) features, and gray-level co-occurrence matrix features to train an ANN.

The performance measures such as average accuracy, average sensitivity, and AUC are presented in Tables 4 and 5. For Marin et al. [35], the performance measures were calculated on their vessel segmented outputs available for download. The average accuracy of our method is 0.9450 and 0.9486 for STARE and DRIVE respectively which is comparatively higher than [35] (refer to Tables 6 and 7). We attribute this increase to the use of a superior pre-processing technique and a better shape descriptor which provided an accurate response to retinal vasculature. [33] reported an average accuracy of 0.9606 and 0.9435 for the two databases respectively. But it can be seen that the results are not consistent over different databases, hence making it less suitable for generalization. Also, the AUC reported in [33] is less than the AUC obtained by our method. We have also included unsupervised methods [45] [46] [47] in our comparison to comprehensively analyze the performance of our method.

**Table 6:** Comparative performance measures of other methods on DRIVE database images.

Method		Accuracy	Sensitivity	Specificity	AUC
Supervised	Marin et al. [35] (calculated)	0.9448	0.6936	0.9801	0.9588
	Thangaraj et al. [33]	0.9606	0.8014	0.9753	0.8884
	Our method	0.945	0.6994	0.9811	0.9394
Un-supervised	Roychowdhury et al. [45]	0.9442	0.7305	0.9787	0.9613
	Strisciuglio et al. [46]	0.9442	0.7655	0.9704	0.9614
	Dash et al. [47]	0.9571	0.7417	0.9861	NA

**Table 7:** Comparative performance measures of other methods on STARE database images.

Method		Accuracy	Sensitivity	Specificity	AUC
Supervised	Marin et al. [35] (calculated)	0.9475	0.6652	0.9803	0.9769
	Thangaraj et al. [33]	0.9435	0.8339	0.9536	0.8938
	Our method	0.9486	0.6298	0.9839	0.9507
Un-supervised	Roychowdhury et al. [45]	0.9332	0.7552	0.9719	0.9617
	Strisciuglio et al. [46]	0.9496	0.7763	0.9695	0.9555

Earlier methods have been able to achieve good accuracy because of them being able to extract thicker and more prominent blood vessels accurately but, their output lacks a majority of thinner blood vessels. Our method, while having comparable accuracy and sensitivity, can detect a majority of thinner blood vessels. The small amount of increase in accuracy and sensitivity is because thin blood vessels, mostly being of one-pixel width occupy a very small portion of the total number of pixels to be classified in the testing set of both databases. Also, because manual segmentation was used to generate the gold standard images, thin blood vessels in the vasculature are prone to manual errors [3].

## Limitations

1. Our method fails to accurately detect very tiny blood vessels of a width of around 1-2 pixels wide which require computation of Higher-order Zernike moments that provide more details.
2. There is a loss of information on the very tiny blood vessels when the morphological operators are applied as few of their pixels tend to blend into the background.
3. Calculation of higher-order Zernike moments are computationally more expensive to calculate and hence difficult to incorporate into our feature set generation as it will become a major bottleneck.
4. Further, the current model computes 36 Zernike moments for generating Dominant Zernike (DM) moments and Sum of Residual Zernike moments and hence incorporating even more Zernike features of higher order will directly impact the computation speed of the model.

## Conclusion and future work

Ophthalmologic disorders can be detected at an early stage by inspection of the vasculature in retinal images. A technique for automatic extraction of blood vessels from the digital color fundus images would make it easy to observe the vasculature and detect pathologies. The proposed method, with improved preprocessing, selection of dominant Zernike Moment based features and hand-selected training points, promises to extract more detailed vasculatures from the given Digital Fundus Images. Since the proposed method can extract the vasculature of thinner blood vessels better than [35], it could be readily used for the screening of ophthalmologic diseases, specifically Diabetic Retinopathy where the first manifestations called microaneurysms, are mainly observed on thinner blood vessels. The improved accuracy and sensitivity over similar supervised methods prove the superiority of the developed method.

In the future, a connectivity-based algorithm for post processing can enhance the connectivity of the detected vasculature and thereby improving the accuracy. Also, Zernike Moment based features look promising as shape descriptors and can be readily used as features for detection of other shape-based pathologies or identifying structures like the Optic Disk and Macula in Digital Fundus Images.
